# Effect of multicomponent exercise intervention on older adults with mild cognitive impairment based on HAPA-TPB theory (MIND-STEP): trial design and baseline data for a randomized controlled trial

**DOI:** 10.1186/s13063-026-09640-4

**Published:** 2026-03-23

**Authors:** Na Zhang, Chenlu Hong, Jiaxiu Sheng, Sisi Yao, Jiali Xu, Qiuxia Wang, Jun Li, Yinke Lu, Haiqin Chen, Yanan Luo

**Affiliations:** 1 Huzhou Third Municipal Hospital, the Affiliated Hospital of Huzhou University, Huzhou, Zhejiang Province China; 2 Department of Global Health, School of Public Health, Peking University, Beijing, China; 3 Huzhou Rehabilitation Hospital, Huzhou, Zhejiang Province China; 4 Institute for Global Health and Development, Peking University, Beijing, China; 5 State Key Laboratory of Vascular Homeostasis and Remodeling, Peking University, Beijing, China

## Abstract

**Background:**

Physical exercise has emerged as a promising non-pharmacological approach for improving cognitive and physical function in older adults with mild cognitive impairment (MCI). However, most existing interventions in nursing homes lack a strong theoretical foundation, standardized delivery, and evidence-based practice. This study aims to develop and evaluate a multicomponent exercise program grounded in the Health Action Process Approach-Theory of Planned Behavior theory (HAPA-TPB) to improve adherence and cognitive outcomes in older adults with MCI living in nursing homes.

**Methods:**

This is a randomized, single-blind, parallel-group controlled trial conducted in nursing homes in Huzhou, China. A total of 156 older adults with MCI were randomly assigned to either a 12-week, group-based multicomponent exercise intervention or a control group receiving usual care. The intervention, theoretically grounded in an integrated HAPA-TPB framework, integrates aerobic, resistance, and mind–body training with structured behavioral change strategies designed to enhance motivation, adherence, and sustained engagement, including peer interaction. The primary outcome is cognitive function assessed by the Montreal Cognitive Assessment-Basic (MoCA-B). Secondary outcomes include physical performance, sleep quality, intrinsic capacity, frailty, social support, and intervention feasibility.

**Results:**

A total of 1351 participants completed preliminary eligibility screening, of whom 156 were eligible and randomized (78 per group). The mean (SD) age was 69.19 years (SD = 8.97), and 69.23% of participants were female. Baseline characteristics were comparable across groups in terms of demographic, socioeconomic, and health status indicators. Baseline cognitive function, assessed using the MoCA-B, showed no significant between-group difference (intervention: mean = 17.29, SD = 2.32; control: mean = 16.74, SD = 2.19; *F* = 2.320, *p* = 0.130), indicating comparability prior to the intervention.

**Discussion:**

This MIND-STEP study will provide high-quality evidence on the effectiveness and implementation of a theory-based, multicomponent exercise intervention for older adults with MCI in nursing homes. Findings will inform future intervention design and policy development to promote cognitive and functional health in institutionalized older populations.

**Trial registration:**

Chinese Clinical Trial Registry ChiCTR2400088301. Registered on 15 August 2024.

**Supplementary Information:**

The online version contains supplementary material available at 10.1186/s13063-026-09640-4.

## Introduction

Dementia is a growing global public health challenge, with over 55 million older adults affected worldwide in 2021 and projections reaching 78 million by 2030 [1]. Despite extensive research efforts, there is currently no cure for dementia, and available pharmacological treatments only offer limited benefits in delaying disease progression. Mild cognitive impairment (MCI), an intermediate stage between normal cognitive aging and dementia, has emerged as a critical window for early intervention. Each year, 5% to 20% of individuals with MCI convert to dementia, and nearly two-thirds of those diagnosed with dementia had previously experienced MCI [2]. Intervening during this transitional phase may therefore provide an opportunity to delay or prevent further cognitive decline. China bears a particularly large burden of MCI. Approximately 15.5% of adults aged 60 years or older are estimated to have MCI, amounting to over 38 million individuals [3], with an annual conversion rate to dementia of approximately 6% [4]. Rapid population aging, uneven distribution of healthcare resources, and increasing demand for long-term care services further amplify the urgency of identifying scalable and effective preventive strategies in this context.

The World Health Organization (WHO) has emphasized the importance of global action to address cognitive decline and dementia, advocating for national dementia plans by 2025 that prioritize prevention, development of disease-modifying therapies, and improved healthcare services. Given the limitations and potential side effects of pharmacological treatments, non-pharmacological interventions such as physical activity are increasingly recognized for their role in enhancing quality of life and reducing the risk of cognitive decline. Evidence suggests that physical exercise, as a low-cost, accessible, and safe intervention, can improve cognitive function and daily living abilities in both cognitively healthy individuals [5–8] and those with MCI [9, 10]. Exercise rehabilitation, rooted in kinesiology, biomechanics, and neurodevelopmental principles, is a well-established method to restore physical and cognitive functions [11, 12] and is considered an effective complementary therapy for individuals with cognitive impairment [13].

Research in neurorehabilitation has rapidly expanded in recent years, with increasing applications among older adults with MCI to improve daily functioning, physical and mental health, quality of life, and social engagement [14, 15]. International research on MCI interventions has shown promising results. For instance, the EXERT trial in the USA and multicomponent exercise studies in Australia and Canada demonstrated that even low- to moderate-intensity exercise could help maintain or improve cognitive function in older adults with MCI [16]. The Finnish FINGER study further confirmed the effectiveness of multidomain lifestyle interventions, including exercise, cognitive training, nutritional guidance, and cardiovascular risk management, as effective strategies to preserve cognitive function [17]. Building on these insights, the World-Wide FINGERS (WW-FINGERS) network has launched global efforts to adapt and implement evidence-based models across various countries, including older adults in China [18].

In China, long-term care institutions such as nursing homes are playing an increasingly important role in supporting the health and well-being of older adults, particularly those with cognitive or functional limitations. These institutional settings offer structured environments, trained staff, and opportunities for supervised group activities—making them a promising platform for the delivery of multicomponent exercise interventions. However, implementation challenges remain, including a lack of standardized intervention protocols, limited behavioral theory integration, insufficient outcome evaluation, and inconsistent adherence among participants. Furthermore, most existing exercise programs in Chinese nursing homes have not been rigorously evaluated through randomized controlled trials, and few are grounded in behavioral change theories that target long-term habit formation.

To address the gaps in theory-driven and systematically evaluated interventions in nursing home settings, the current MIND-STEP study aims to develop and test a multicomponent group-based exercise program for older adults with MCI residing in nursing homes. The intervention is grounded in the integrated Health Action Process Approach and Theory of Planned Behavior (HAPA-TPB) framework, which is designed to enhance motivation, promote adherence, and support sustained behavior change. The program incorporates aerobic, resistance, and mind–body training components, alongside structured behavioral strategies such as goal-setting, action planning, and social support facilitation. A randomized controlled trial will be conducted to assess the effectiveness and feasibility of the intervention, providing high-quality evidence to inform future implementation and policy development in aging care in China.

## Methods

The protocol is written following the Standard Protocol Items: Recommendations for Interventional Trials (SPIRIT) reporting guidelines [19–21] and in the TiDIER format [22]. A completed SPIRIT checklist is provided in the Supplementary Table S1.

### Study design, setting, and participants

**Fig. 1:**
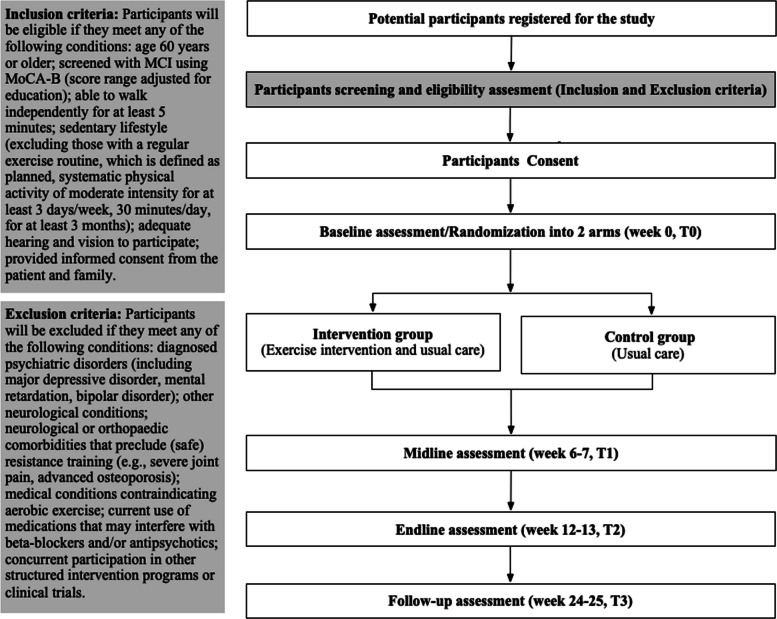
Flow chart

This MIND-STEP study is a superiority, single-blind, parallel-group randomized controlled trial designed in a 1:1 ratio to evaluate the effectiveness of a multicomponent exercise intervention among MCI older adults residing in a nursing home in China. The trial design and timeline are summarized in Fig. 1 and Table 1. Ethics approval was granted by the Medical Ethics Committee of Huzhou Third People’s Hospital and was registered (trial registration number ChiCTR2400088301). Participants will be randomly assigned in a 1:1 ratio to either an intervention group receiving 12 weeks of a multicomponent exercise program or a control group receiving usual care with no structured exercise intervention. Assessors responsible for performing the baseline (week 0), midline (week 6–7), endline (week 12–13), and follow-up assessments (weeks 24–25, corresponding to approximately 12–13 weeks post-intervention) will be blinded to allocation.

**Table 1:**
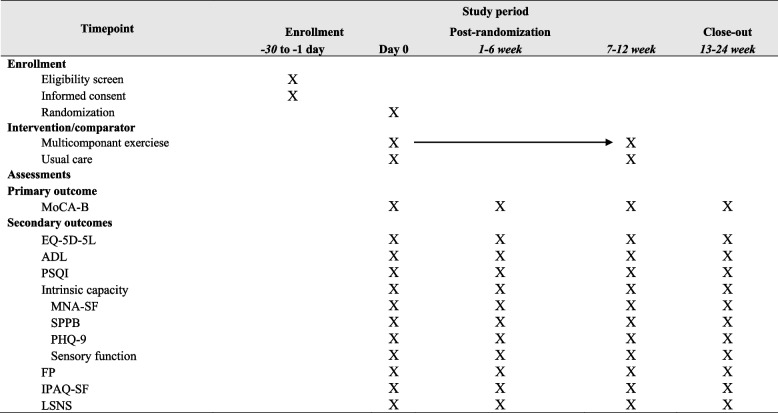
Participant timeline: schedule of enrollment, interventions, and assessments—the MIND-STEP randomized trial

*MoCA-B *Montreal Cognitive Assessment-Basic (Chinese version), *EQ-5D-5L *EuroQol-5D-5L, *ADL *Activity Daily of Life, *PSQI *Pittsburgh Sleep Quality Index, *MNA-SF *Mini Nutritional Assessment–Short Form, *SPPB *Short Physical Performance Battery, *PHQ-9 *Patient Health Questionnaire-9, *FP *frailty phenotype, *IPAQ-SF *International Physical Activity Questionnaire–Short Form, *LSNS *Lubben Social Network Scale

Eligible participants will be older adults residing in nursing homes aged 60 years or above who do not engage in regular physical exercise. Regular exercise will be defined as participating in planned and structured moderate-intensity physical activity for at least 30 min per session, on a minimum of 3 days per week, consistently over the past 3 months. Additional inclusion and exclusion criteria are presented in Fig. 1. The latest protocol (version 1.0) was dated 25 July 2024.

### Patient and public involvement

None.

### Recruitment and eligibility

Recruitment began in October 2024 at nursing homes in Huzhou city, and is anticipated to conclude by March 2025. Prospective participants will be screened by the clinical team, and clinical records will be reviewed to confirm eligibility. Suitable older adults will be approached face-to-face during routine care visits by trained research staff. Posters will be displayed at the senior care organizations, allowing potential participants to contact the study team if they are interested. Adults 60 years and above with MCI will be eligible for inclusion in the trial. To identify participants with MCI, the Montreal Cognitive Assessment-Basic (MoCA-B) will be used, with cut-off scores adjusted to account for differences in educational attainment. Specifically, participants will be considered eligible for inclusion if their MoCA-B scores fall within the following ranges: 16 to 24 for those with more than 12 years of education; 15 to 22 for those with 7 to 12 years of education; and 13 to 19 for those with 6 years of education or less. To further correct for educational and literacy-related bias, an additional point will be added to the total score for participants with fewer than 4 years of education, and one additional point will also be added for those identified as illiterate, regardless of formal education level. The maximum possible score on the MoCA-B is 30. The inclusion criteria and exclusion criteria are presented in Fig. 1.

### Randomization and blinding

Participants were randomly assigned to either the intervention or control group in a 1:1 ratio using a computer-generated randomization sequence. The allocation sequence was generated in advance by an independent researcher who is not involved in participant recruitment, outcome assessment, or data analysis. Allocation concealment was implemented using a centrally maintained randomization list held by a designated study supervisor independent of recruitment and assessment procedures. The allocation list was not accessible to recruiters or outcome assessors. After eligibility confirmation and completion of baseline assessments, participants were assigned sequentially according to the predefined randomization list in the order of enrollment. Group allocation was disclosed only after baseline assessments were completed.

Due to the nature of the behavioral intervention, participants and intervention providers cannot be blinded to group allocation. However, outcome assessors will remain blinded to group allocation throughout the study to minimize assessment and analytical bias. Outcome assessors will not have access to the randomization list and will be instructed to avoid any discussions with participants regarding their assigned groups. Participants will also be reminded not to disclose their group allocation during assessments. Unblinding will be permitted only in cases of medical emergencies where knowledge of the participant’s allocated intervention is essential for patient safety. The procedure for unblinding involves contacting the principal investigator or designated study physician, who will access the allocation information from the secure trial database and document the reason for unblinding.

### Intervention

#### Intervention group

Participants in the intervention group will receive a 12-week, group-based multicomponent exercise program to be delivered through in-person, face-to-face sessions. Each session will last 30 min and will be conducted twice per week. The multicomponent exercise intervention will consist of five structured components: a warm-up, aerobic training, resistance training, mind–body training, and a cool-down period, forming a complex, theory-informed approach tailored to the physical and cognitive needs of older adults. Participants will be guided by nurses in the nursing home to perform aerobic training, resistance training, and dual-task (mind–body) training throughout the 30-min session. A Rate of Perceived Exertion (RPE) of 5–6, corresponding to moderate intensity, will be recommended. Core aerobic exercises will include shoulder movements, hip abduction or extension, and thigh lifts. Resistance training will consist of functional movements such as squats and trunk twists. Mind–body training will incorporate coordinated activities such as seated marching combined with attention tasks and standing marching paired with inhibition tasks. All participants will be instructed to complete a brief warm-up at the beginning and a cool-down routine at the end of each session to promote safety and recovery.

The intervention will feature a group-based multicomponent exercise program and will be conceptually grounded in the integrated HAPA-TPB model. This theoretical framework was selected to address known facilitators and barriers to exercise engagement among older adults [23], as it incorporates both the motivational phase (e.g., intention formation) and the intenders phase (e.g., planning, execution, maintenance of behavior) and action phase, thereby providing a comprehensive framework for promoting sustainable health behavior change in later life. This integrated model combines the stage-based behavioral pathway of HAPA (preintenders, intenders, and actors) [24, 25] with key motivational determinants from TPB, including attitudes, subjective norms (operationalized as social support in this study), and perceived behavioral control [26]. Particular emphasis was placed on enhancing self-efficacy and peer-based social support as core mechanisms to strengthen adherence and sustain exercise behavior.

Within this framework, exercise behavior change was structured as a phased process. In the preintender phase (intention formation), intervention strategies were designed to enhance task self-efficacy through baseline assessments, clear explanation and demonstration of exercise components, strengthen subjective norms and social support via peer modeling and group-based participation, and promote positive exercise attitudes through education and benefit framing. During the intender phase (transition from intention to action), action and coping planning were operationalized through structured scheduling, barrier identification, and guided problem-solving. Task progression charts and early mastery experiences were incorporated to improve maintenance self-efficacy and reinforce perceived behavioral control. In the actor phase (action and maintenance), strategies aimed to strengthen recovery self-efficacy while sustaining social support. Reassurance and structured re-engagement were provided to participants who encountered difficulties, with facilitator check-ins normalizing setbacks. Safety monitoring and individualized exercise modifications were implemented to reduce perceived risk. Ongoing encouragement from instructors, peer interaction, and support from nursing home staff reinforced long-term adherence. Collectively, these strategies were designed to mitigate common barriers to sustained engagement, such as motivational decline and emotional apathy among older adults with MCI, and to promote long-term behavioral adherence. The theoretical constructs (e.g., self-efficacy) will be used solely to guide intervention development and delivery and will not be formally measured as study outcomes in the present trial. A detailed mapping of theoretical constructs to specific intervention components is presented in Table 2.

**Table 2:** Adherence-enhancing and health behavior change techniques in a 12-week multicomponent exercise intervention for older adults with MCI based on HAPA-TPB Theory

Intervention phase	Intervention objective	Plan for group-based intervention in nursing homes
**Preintender** (behavioral intention formation)	Enhance task self-efficacy	Prior to the intervention (week 0), conduct baseline assessments of physical and cognitive function. Supervisors provide participants with a clear explanation of the group-based exercise prescription and demonstrate core movements (e.g., aerobic, resistance, mind–body training)
	Enhance outcome expectations	During the introductory session (week 0), participants are informed of potential health benefits, such as improved physical function and cognition. Verbal encouragement and group-based goal tracking are introduced to reinforce progress. Small rewards (e.g., certificates, recognition in group meetings) are given based on attendance and effort
	Enhance subjective norms	At baseline, participants are introduced to peer role models and encouraged to train alongside others in their nursing home. Staff highlight the social benefits and collective identity of participating in the program. Group norms are reinforced by praise and mutual encouragement
	Enhance social support	Sessions are facilitated by a consistent instructor to build trust. Nursing home staff are encouraged to support participants. Peer support is fostered through shared exercises and group discussion
	Enhance attitudes	Educational sessions are held at the start of the intervention, featuring short talks or videos on the benefits of exercise for aging and cognitive health. These are followed by Q&A and small-group reflections to reinforce positive attitudes
**Intender** (from preintenders to intenders)	Action planning	Supervisors work with participants to plan when and how they will attend twice-weekly group sessions. Participants receive a printed plan outlining the frequency, intensity, duration, and type of exercise based on the FITT-VP principle
	Coping planning	Instructors discuss common barriers to participation (e.g., fatigue, joint pain, scheduling) and guide participants through generating coping strategies. Group sharing and encouragement are integrated into each session
	Improve maintenance self-efficacy	Participants are reminded of their ability to maintain training through positive reinforcement, feedback, and group support. Progress charts are displayed to visualize adherence
**Actor (action)**	Enhance recovery self-efficacy	For participants who miss sessions or experience difficulty, instructors provide reassurance and strategies for re-engagement. Group discussion and facilitator check-ins help normalize setbacks
	Reduce risk perception	Facilitators proactively address safety concerns and physical discomfort. Stretching routines, monitoring of vital signs, and individualized modifications are provided to reduce fear of injury
	Enhance social support	Support is provided at multiple levels: from instructors (weekly encouragement), peers (in-session interactions), and nursing home staff (logistical and motivational support). Group-based formats foster peer encouragement, accountability, and shared commitment

#### Control group

Participants in all study groups, including the control group, will receive routine nursing home care unrelated to structured exercise or cognitive training. Usual care will encompass three main components: (1) *basic care*, maintaining a clean, well-ventilated, and tidy living environment; assistance with personal hygiene (e.g., bathing and clothing changes twice per week); and toileting support for individuals with incontinence or limited mobility; (2) *nutritional care*, provision of three meals per day according to the nursing home’s standard schedule, with feeding assistance provided as needed; and (3) *safety care*, participants remain in designated indoor areas, and assistive devices are provided when necessary to ensure safe mobility.

#### Concomitant care

Participants in all study groups will be permitted to continue their usual medical care and prescribed medications throughout the trial period. Routine nursing home care unrelated to structured exercise or cognitive training will continue as standard practice. During screening, individuals currently enrolled in other interventional research studies or those with current use of medications that may compromise safe participation in the exercise program (e.g., beta-blockers or antipsychotics) will be excluded according to the eligibility criteria.

### Measurements

#### Primary outcome measures

All outcome data will be collected through a face-to-face electronic questionnaire system administered by trained physicians or nurses who have received standardized training. The primary outcome is cognitive function measured by the Montreal Cognitive Assessment-Basic (MoCA-B) score. The MoCA-B is a validated screening tool specifically developed to detect mild cognitive impairment (MCI) in individuals with limited educational backgrounds or illiteracy [27, 28]. It evaluates a range of cognitive domains, including executive function, immediate and delayed recall, verbal fluency, orientation, calculation, abstraction, visuoperception, and naming. The assessment takes approximately 15 min to complete and is scored out of a maximum of 30 points. To reduce potential bias related to education and literacy, one point will be added to the total score for participants with fewer than 4 years of education or those identified as illiterate (defined as the inability to read or write fluently in daily life), provided their raw score is below 30.

MoCA-B will be assessed at baseline, 6 weeks, 12 weeks, and 24 weeks post-randomization. The primary endpoint is defined as the between-group difference in change from baseline to 12 weeks post-randomization (end of the intervention period). Results will be presented as adjusted mean differences with 95% confidence intervals. All primary analyses will follow the intention-to-treat principle.

#### Secondary outcome measures

Secondary outcomes include Quality of Life measured by the EuroQol-5D-5L (EQ-5D-5L), Activities of Daily Living (ADL), Pittsburgh Sleep Quality Index (PSQI) [29], intrinsic capacity, frailty, social support, and intervention cost and benefit, which have been validated among Chinese older adults. Intrinsic capacity will be evaluated in accordance with the WHO Integrated Care for Older People (ICOPE) guidelines [30], across the following domains: cognitive capacity assessed by MoCA-B, vitality assessed using Mini Nutritional Assessment–Short Form (MNA-SF), locomotion measured by Short Physical Performance Battery (SPPB), psychological status assessed using Patient Health Questionnaire (PHQ-9) [31], and sensory determined by self-reported vision and hearing questions. Frailty will be assessed using the frailty phenotype (FP) [32, 33], physical activity levels will be evaluated using the International Physical Activity Questionnaire–Short Form (IPAQ-SF), and social support will be assessed by the Lubben Social Network Scale (LSNS) [34].

Secondary outcomes will be assessed at baseline, 6 weeks, 12 weeks, and 24 weeks post-randomization. Secondary endpoints will be defined as the between-group differences in change from baseline at each post-randomization timepoint. Results will be reported as adjusted mean differences or risk differences with corresponding 95% confidence intervals, as appropriate to the outcome type. All secondary analyses will follow the intention-to-treat principle.

#### Process evaluation

A comprehensive process evaluation will be conducted to assess the quality of intervention implementation and its potential impact on study outcomes. This evaluation will address the following dimensions: Eligibility and recruitment will be tracked using data on the proportion of eligible participants, recruitment and retention rates, and reasons for drop-off. Intervention acceptability will be evaluated through supervisory documents, adherence records and satisfaction surveys [35]. Adherence will be measured based on the number of dropouts and session frequency across groups. Participants will be classified as having dropped out if they meet any of the following criteria: average training frequency of ≤ 2 times per week over 3 weeks; average frequency of ≤ 1 time per week for two consecutive weeks; or no sessions completed within a 1-week period. Temporary absences of 1–2 weeks due to medical treatment or personal reasons will be exempt if participants resume full participation thereafter. All dropouts will be encouraged to complete follow-up assessments.

Outcome acceptability will be assessed by calculating the completion rates of outcome questionnaires at baseline, 6 weeks, and 12 weeks. Adverse events, including falls, fractures, muscle strains, cardiovascular events, and new or exacerbated musculoskeletal, cardiovascular, or metabolic conditions, will be monitored throughout the study. Participants in the intervention group will be asked about any adverse events following each session. All reported events will be documented and the responsible authorities will be notified promptly, and relevant free examinations and management strategies will be provided within the nursing home or referred to the appropriate medical institution. Participants experiencing minor adverse events may continue with the study if the participants’ condition does not worsen and if adequate rest and treatment do not interfere with ongoing participation.

### Sample size estimation

The sample size was calculated using PASS 2021 software (version 21.0.3). A two-sample independent *t*-test based on effect size was used for estimation, with cognitive function as the primary outcome. Previous studies have reported effect sizes of exercise interventions on cognitive improvement ranging from 0.54 to 2.85 [36–39]. To ensure a conservative estimation, an effect size of 0.54 for cognitive improvement was selected. With a two-sided significance level of *α* = 0.05 and statistical power of 85% (1 − *β* = 0.85), a minimum of 126 participants (63 per group) was required. To account for an anticipated 20% attrition rate, the planned recruitment target was set at 160 participants (80 per group). Due to unavoidable circumstances, such as relocation or death during the recruitment period, 156 participants were ultimately enrolled, which remained sufficient to meet the predefined statistical power requirements.

### Data collection and management

The trial will be coordinated by the Huzhou Third Municipal Hospital, the Affiliated Hospital of Huzhou University, which is responsible for overall study management and coordination across study sites. Data management will be conducted by trained research staff under the supervision of the principal investigator at Peking University. An independent data monitoring committee and endpoint adjudication committee will not be established due to the low-risk nature of the behavioral intervention; however, study progress, protocol adherence, and data quality will be regularly reviewed by the core research team. Any protocol deviations or adverse events will be documented and discussed in regular team meetings. Data quality will be ensured through double-entry and regular audits. Personal data will be kept confidential, and no personal information will be reported.

### Statistical analysis plan

Statistical analyses will be undertaken by an independent statistician, according to a statistical analysis plan developed and signed before database lock. All analyses will use STATA/MP 17.0 or a later version if available.

#### Summary of trial flow and baseline characteristics

Participant flow through the trial will be documented using a CONSORT diagram. The baseline characteristics will be described using summary statistics. Baseline demographic and clinical characteristics will be summarized by group and overall. Continuous variables will be described using means and standard deviations (SD) or medians and interquartile ranges (IQR), as appropriate, while categorical variables will be presented as frequencies and percentages. Baseline values for each outcome variable will be reported at different time points. Comparisons between participants who complete follow-up and those lost to follow-up will be conducted using independent t-tests and chi-square tests to evaluate potential bias.

#### Analysis of the primary outcome and secondary outcomes

The primary outcome (MoCA-B score) will be analyzed using linear mixed-effects models (LMM) within a Difference-in-Differences (DID) framework. All available time points (baseline, week 6, week 12, and week 24) will be included in the primary LMM analysis. Fixed effects will include group, time, and the group-by-time interaction, along with relevant baseline covariates (e.g., age and sex). The DID estimate corresponds to the group-by-time interaction term at week 12, representing the between-group difference in change from baseline to week 12 (the primary endpoint). Short-term (baseline to week 6), long-term (baseline to week 24), and durability effects (week 12 to week 24) will also be explored using the same framework. For secondary outcomes, continuous variables will be analyzed using similar linear mixed-effects models. Binary outcomes will be analyzed using generalized estimating equations (GEE) with a binomial family and logit link. Marginal predicted probabilities will be used to derive risk differences between groups. All analyses will be performed in the intention-to-treat (ITT) population.

Missing outcome data will be handled under the missing-at-random assumption using LMM models, which inherently accommodate incomplete repeated measurements. In addition, a sensitivity analysis will be conducted using multiple imputation with chained equations to assess the robustness of the primary results. Exploratory analyses for all outcomes will also be performed using per-protocol analyses to evaluate efficacy among participants who complete the intervention as specified and provide both baseline and 12-week outcome data. Subgroup analyses will be conducted to explore potential heterogeneity of treatment effects. Two-sided *p*-values of less than 0.05 will be considered to indicate statistical significance.

### Trial monitoring

Trial conduct will be monitored by the core research team to ensure adherence to the study protocol, data quality, and participant safety. Monitoring activities will include regular review of recruitment progress, participant adherence, data completeness, and documentation of adverse events. Monitoring will be conducted on a monthly basis through team meetings and periodic review of study records. Any protocol deviations will be documented and discussed within the research team. Serious adverse events will be reported promptly to the institutional ethics committee in accordance with regulatory and institutional requirements. Decisions regarding potential trial modification or termination will be made by the principal investigator in consultation with the ethics committee. Given the low-risk nature of this behavioral exercise intervention, an independent data monitoring committee has not been established, and no formal interim analyses or predefined stopping guidelines are planned.

## Results

### Baseline characteristics of randomized participants

**Fig. 2:**
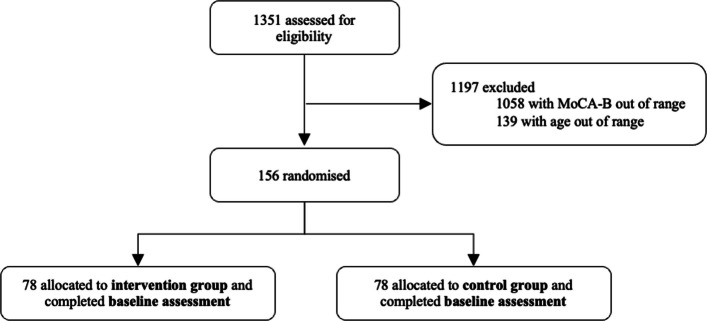
Trial profile

A total of 1351 participants completed preliminary eligibility screening. Based on MoCA-B results, 156 participants met the inclusion criteria and were subsequently randomized into either the intervention group (*n* = 78) or the control group (*n* = 78) and baseline assessments (week 0) were completed **(**Fig. 2**).**

Baseline characteristics are presented in Table 3. A total of 156 participants were randomized to either the exercise intervention group (*n* = 78) or the control group (*n* = 78). The mean age of the total sample was 69.19 years (SD = 8.97), with a higher proportion of female participants (*n* = 108, 69.23%) than male participants (*n* = 48, 30.77%). The two groups were generally well-balanced at baseline, with no statistically significant differences observed in age, gender, marital status, educational attainment, Hukou status, occupation and monthly income.

**Table 3:** Baseline characteristics of participants

**Variable**	**Intervention group** (*N* = 78)	**Control group** (*N* = 78)	*χ*^2^/*F*	*P* value
**Age (year)**	68.63(8.61)	69.76(9.35)	0.610	0.434
**Sex**			0.120	0.729
Male	23 (29.49)	25 (32.05)		
Female	55 (70.51)	53 (67.95)		
**Marital status**			0.027	0.869
Married or partnered	48 (61.54)	49 (62.82)		
Other	30 (38.46)	29 (37.18)		
**Educational attainment**			0.026	0.872
Primary school and below	42 (53.85)	43 (55.13)		
High school and above	36 (46.15)	35 (44.87)		
**Hukou**			0.103	0.748
Urban	37 (47.44)	35 (44.87)		
Rural	41 (52.56)	43 (55.13)		
**Occupation**			2.136	0.711
Government or institutional staff	7 (8.97)	4 (5.13)		
Skilled professional/technical personnel	6 (7.69)	6 (7.69)		
Agricultural workers	10 (12.82)	14 (17.95)		
Others	47 (60.26)	43 (55.13)		
Unemployed or not currently working	8 (10.26)	11 (14.10)		
**Household income per month**			0.045	0.978
Low income (tertile 1)	28 (35.90)	28 (35.90)		
Middle income (tertile 2)	33 (42.31)	34 (43.59)		
High income (tertile 3)	17 (21.79)	16 (20.51)		

Continuous variables are reported as mean ± SD and analyzed using ANOVA; categorical variables are reported as frequency (%) and analyzed using chi-square tests

Table 4 presents the baseline health status of participants. No significant between-group differences were detected across a range of health indicators. The health status was generally comparable between groups. Baseline values for self-rated health, MNA-SF, SPPB, PHQ-9, PSQI, ADL, frailty, social support situation, and MoCA-B were also well-balanced. Although self-rated health scores were slightly higher in the intervention group, the control group reported marginally poorer sleep quality. Cognitive function, assessed by the MoCA-B, was also similar between groups, with mean scores of 17.29 (SD = 2.32) in the intervention group and 16.74 (SD = 2.19) in the control group. Overall, these findings indicate that both randomized groups were comparable at baseline in terms of both demographic characteristics and health status, providing a sound basis for evaluating the intervention effects.

**Table 4:** Baseline health status of participants

**Variable**	**Intervention group** (*N* = 78)	**Control group** (*N* = 78)	*χ*^2^/*F*	*P* value
**Self-rated health, mean (SD)**	71.76 (9.00)	69.50 (8.33)	2.670	0.104
**Nutritional status (MNA-SF)**			1.378	0.502
Normal nutritional status	24 (30.77)	26 (33.33)		
At risk of malnutrition	47 (60.26)	41 (52.56)		
Malnourished	7 (8.97)	11 (14.10)		
**SPPB, mean (SD)**	7.30 (2.75)	6.96 (2.86)	0.540	0.463
**Depression status (PHQ-9)**			0.289	0.591
No	58 (74.36)	55 (70.51)		
Yes	20 (25.64)	23 (29.49)		
**PSQI, mean (SD)**	11.95 (4.62)	13.46 (7.45)	2.320	0.130
**Distance vision**			2.937	0.087
Good	6 (7.69)	13 (16.67)		
Poor	72 (92.31)	65 (83.33)		
**Near vision**			6.798	0.009
Good	2 (2.56)	11 (14.10)		
Poor	76 (97.44)	67 (85.90)		
**Hearing**			0.880	0.348
Good	7 (8.97)	4 (5.13)		
Poor	71 (91.03)	74 (94.87)		
**ADL score, mean (SD)**	21.32 (7.08)	22.31 (7.58)	0.710	0.402
**Frailty status (FP)**			1.354	0.245
No	61 (78.21)	65 (83.33)		
Yes	17 (21.79)	13 (16.67)		
**Limited social network**			0.104	0.747
No	35 (44.87)	33 (42.31)		
Yes	43 (55.13)	45 (57.69)		
**Cognitive status**				
**MoCA-B score, mean (SD)**	17.29 (2.32)	16.74(2.19)	2.320	0.130

*MNA-SF *Mini Nutritional Assessment–Short Form, *SPPB *Short Physical Performance Battery, *PHQ-9 *Patient Health Questionnaire-9, *PSQI *Pittsburgh Sleep Quality Index, *MoCA-B *Montreal Cognitive Assessment-Basic (Chinese version). Continuous variables are reported as mean ± SD and analyzed using ANOVA; categorical variables are reported as frequency (%) and analyzed using chi-square tests

## Discussion

This protocol outlines a rigorously designed randomized controlled trial aiming to evaluate the effects of a 12-week, theory-informed multicomponent exercise intervention on cognitive and physical function among older adults with MCI residing in nursing homes in China. Given the growing aging population and increasing prevalence of MCI in institutionalized settings, there is an urgent need for effective, scalable interventions that address both physical and cognitive health. This MIND-STEP study seeks to address a key evidence gap by implementing a structured program grounded in the integrated HAPA-TPB, combining aerobic, resistance, and mind–body exercises with behavioral strategies to promote adherence and long-term engagement.

The baseline characteristics of the study cohort demonstrated that participants were representative of the broader population of older adults with MCI in residential care, with a mean age of 69.19 years and a predominance of female participants, consistent with demographic trends in nursing homes. Importantly, no significant between-group differences were observed across demographic, socioeconomic, or health-related variables, suggesting that the randomization process was successful and that the two arms are comparable at baseline. This provides a robust foundation for assessing intervention effects on subsequent outcomes. A key strength of this trial lies in its integration of behavioral theory into the intervention framework. By embedding motivational and volitional strategies derived from the HAPA-TPB model, the program is designed not only to enhance short-term participation but also to foster long-term behavior change. The group-based delivery format, coupled with tailored support strategies such as action planning, social reinforcement, and coping strategies, is expected to improve feasibility and acceptability in nursing home settings. Nonetheless, several potential limitations warrant consideration. First, as the trial is conducted in nursing homes within a single urban region, generalisability to community-dwelling older adults or those in rural areas may be limited. Second, while the follow-up period captures short- to mid-term effects, longer-term sustainability of intervention benefits remains to be evaluated. Third, although efforts have been made to blind outcome assessors and standardize procedures, some risk of performance or detection bias cannot be fully excluded. Finally, repeated administration of the MoCA-B may introduce practice effects, which could partially inflate cognitive scores over time. Although both groups were assessed at identical time points and the primary analysis focused on between-group differences, residual practice effects cannot be fully excluded.

In conclusion, this trial will contribute novel evidence on the effectiveness and implementation of a structured, multicomponent exercise intervention for older adults with MCI, supported by behavioral theory and delivered in a nursing home setting. The findings will inform the development of integrated care strategies targeting cognitive and physical health in aging populations and may support broader adoption of exercise-based interventions within long-term care services in China and beyond.

## Supplementary Information

Supplementary Material 1. Informed consent.

Supplementary Material 2: Table: Appendix: All items from the World Health Organisation Trial dataset.

Supplementary Material 3.

## Data Availability

Access to de-identified data may be granted to interested parties 12 months after the publication of the principal paper addressing primary research questions, upon request via email to the responsible person (luoyanan@bjmu.edu.cn).
