# Supplementary material for: Effect of multicomponent exercise intervention on older adults with mild cognitive impairment based on HAPA-TPB theory (MIND-STEP): trial design and baseline data for a randomized controlled trial
Source: Trials. 2026 Mar 23;27:342. doi: 10.1186/s13063-026-09640-4 (PMC13134337; doi:10.1186/s13063-026-09640-4)
Supplement: Supplementary file 1 — Supplementary Material 1. Informed consent. [file 13063_2026_9640_MOESM1_ESM.pdf]

DeepL

Subscribe to DeepL Pro to edit this document.
Visit www.DeepL.com/pro for more information.

Informed Consent Form

Dear Sir/Madam,

We wish to invite you to participate in a clinical study on brain health promotion for the elderly, conducted in collaboration between our hospital and Peking University Health Science Centre. Before deciding whether to join this study, please read the following information as carefully as possible. It will help you understand the nature of the research, its purpose, and the potential benefits, risks, and discomforts it may entail for you.This study has been reviewed and approved by the Ethics Review Committee of Huzhou Third People's Hospital and the Biomedical Ethics Committee of Peking University. It complies with relevant Chinese regulations and ethical principles protecting research participants' rights, including the Declaration of Helsinki.

1. Research Background and Objectives

Mild cognitive impairment (MCI) represents a transitional stage between normal cognitive ageing and dementia, posing a significant threat to global public health.Annually, 5 – 20% of MCI patients progress to dementia, with nearly two-thirds of dementia cases presenting as MCI. In 2018, approximately 50 million older adults worldwide experienced varying degrees of cognitive impairment, projected to increase to 82 million by 2030.In China, over 20% of older adults exhibit MCI, with approximately 6% of these individuals progressing to dementia annually. Non-pharmacological interventions constitute the primary treatment approach for MCI. Research indicates that exercise programmes can reduce the risk of adverse health outcomes in MCI or dementia patients while positively influencing their functional abilities.Research indicates that multicomponent exercise interventions yield superior cognitive outcomes for elderly individuals with MCI, representing a promising non-pharmacological treatment approach characterised by low risk-cost ratios, flexible intervention schedules, and minimal spatial requirements. This facilitates access to treatment for populations in medically underserved regions. Our study aims to develop a brain health intervention model for elderly individuals with mild cognitive impairment, with the objective of enhancing cognitive function.

2. Who will be invited to participate in this study?

Eligible participants

① Individuals aged 60 years or older;

② Screened as MCI patients using the MoCA scale;

③ Capable of walking independently for over five minutes;

④ Leading a predominantly sedentary lifestyle in daily activities (excluding those with regular exercise habits defined as planned, systematic physical activity of moderate intensity for at least 30 minutes per day, 3 days per week, sustained for a minimum of 3 months)

⑤ Self-reported adequate hearing and visual function;

⑥ Obtain informed consent from both the patient and their family.

Exclusion criteria

① Individuals with comorbid mental disorders (including major depressive disorder, intellectual disability, or bipolar disorder);

② Individuals with other neurological impairments;

③ Individuals with neurological or orthopaedic conditions rendering resistance training unsafe (e.g., significant joint pain, osteoporosis);

④ Contraindications for aerobic exercise

⑤ Use of beta-blockers and/or antipsychotic medications;

⑥ Current participation in another intervention programme.

3. How was the study conducted?

All intervention training sessions occur twice weekly for a total of 24 sessions, with the intervention lasting three months. The intervention is delivered in groups of approximately ten participants, with the control group utilising a placebo control approach. Three complimentary health assessments will be conducted at the start, midway, and completion of the intervention, followed by a follow-up assessment three months post-intervention completion.Should you agree to participate, you will be randomly assigned to either the exercise intervention group or the control group. You will not be able to predict or choose which group you are assigned to.

If you meet the inclusion criteria and consent to participate, you will assist the doctor/nurse with the following procedures: Upon enrolment, you will complete questionnaire surveys assessing physical function, quality of life, fatigue, intrinsic capacity (cognitive function, mobility, vitality, sensory function [vision and hearing], depressive symptoms),frailty, and sleep patterns;Assessments will be conducted at the mid-intervention stage (weeks 6-7), post-intervention (weeks 12-13), and 24-25 weeks post-intervention. You will again complete questionnaire surveys with the doctor regarding physical function, quality of life, fatigue, intrinsic capacity (cognitive function, mobility, vitality,vision and hearing sensation, depressive symptoms), frailty, and sleep status.

4. How long will this study last?

This study will conduct a three-month intervention training programme, with assessments scheduled at weeks 6–7 during the intervention period, weeks 12–13 following intervention completion, and weeks 24–25 post-intervention.

5. What are the risks of participating in this study?

Certain questions during the survey may cause you discomfort; you may decline to answer them.Exercise safety concerns may arise during the intervention. Should you experience discomfort or any of the following conditions rendering continued participation inadvisable: (1) exacerbation of existing medical conditions; (2) occurrence of serious adverse events(e.g., falls, fractures, muscle strains, cardiovascular triggers) during the exercise intervention protocol, you may immediately withdraw from the study. The following risk mitigation measures will be implemented:(1) From a safety perspective, enhance participant selection criteria by implementing exercise assessments and screening to exclude individuals unable to walk independently or with contraindications to exercise. Conduct small-scale safety trials prior to large-scale studies to further validate the feasibility of this exercise intervention;(2) Provide relevant exercise safety education to elderly participants in the exercise intervention (including pre-exercise safety reminders such as appropriate clothing and footwear selection, signs requiring immediate cessation of exercise, safety precautions,and situations unsuitable for exercise);(3) Exercise should comprise three phases: preparation,main activity, and cool-down. Incorporating flexibility exercises at both the start and end reduces risks of cardiovascular incidents and accidental injury; (4) Exercise venues should ideally be quiet, with dry, level surfaces and suitable ambient temperatures;(5) It is recommended to provide tables and chairs at the exercise intervention site for participants who feel fatigued or physically overwhelmed to rest; (6) It is recommended that family carers or relatives accompany participants throughout the exercise intervention to enable prompt intervention in case of accidents (despite the recommended movements having a high safety factor and participants being rigorously selected). Any related costs incurred will be borne by the project team; participants will not be required to cover any expenses.

6. What are the benefits of participating in this study?

This brain health promotion programme aims to enhance cognitive function, sleep quality, physical condition, and quality of life among older adults, thereby preventing the progression of dementia. While we cannot guarantee individual benefits from the programme, participation offers opportunities to establish long-term trust with healthcare professionals and cultivate healthy behavioural habits.

7. Is participation in and completion of this study mandatory?

Participation in this study is entirely voluntary. Should you decline, this will have no adverse impact on your current or future medical care. Even after agreeing to participate, you may withdraw at any time by informing the researchers. Your withdrawal will not affect your access to standard medical services.Upon your withdrawal, the researchers will proactively delete any personal information collected. Your relevant data will be securely stored until final destruction, during which time it will not be further used or disclosed.

8. Regarding Study Costs and Compensation

The primary costs associated with this study include equipment fees, material costs, and travel expenses, all of which are covered by the project team's funding. You will not be charged any fees for participating in this research study at .

9. Handling of Research-Related Injuries

Should any accidental injury occur as a result of implementing the research protocol to achieve the study objectives, we shall provide necessary medical treatment and offer compensation or indemnification in accordance with the law.

VIII. Will participating in this study increase my medical expenses?

You will not be charged any fees for participating in this research study. Therefore, this study will not impose any additional financial burden upon you.

IX. Are there alternative treatment options currently available?

Should you choose not to participate in this study, alternative treatment options, such as medication, remain available. Please consult the investigator or your doctor regarding specific treatment plans.

10. Will my information be kept confidential?

Your research data and personal details are strictly confidential. All electronic records will be identified by a research code rather than your name. No personally identifiable information will be disclosed to individuals outside the research team without your explicit consent.

11. Who should I contact if I have questions or difficulties?

For any questions regarding this study, please contact: Hong Chenlu (13141224025). For matters concerning your rights as a participant, you may contact the Ethics Review Committee of Huzhou Third People's Hospital at 0572-2132467. Contact persons: Wen Qiqing, Wang Jun.

Informed Consent Form · Consent Signature Page

Declaration of Consent

1. I have read this informed consent form, and the responsible personnel involved in the project have provided me with a detailed explanation of the trial's purpose, content, risks, and potential benefits.

2. I have discussed and inquired about matters pertaining to this study, and the answers provided have satisfied me.

3. I have had sufficient time to make my decision.

4. I voluntarily consent to participate in the clinical research described herein.

5. Should I withdraw from the trial for personal reasons, I will promptly inform the doctor of any changes in my medical condition.

6. Should any additional treatment become necessary due to changes in my condition, I shall either consult the doctor beforehand or truthfully inform the doctor afterwards.

7. I consent to representatives from the Medicines Regulatory Authority, Ethics Committee, or funding body accessing my research records.

8. I shall receive a signed and dated copy of this informed consent form.

9. I have truthfully disclosed my medical history. Should any information be withheld, I accept full responsibility for the consequences.

Finally, I consent to participate in this clinical study and undertake to comply with all medical instructions.

Signature of Study Participant: Date:_______ _____ _____

Research Participant Contact Number:

Signature of Legal Guardian: Date:_______ Year_____ Month_____ Day

Relationship to Research Participant: Legal Guardian's Telephone:

I confirm that I have explained the details of this study to the research participant, including their rights and the potential benefits and risks, and have provided them with a signed informed consent form.

Doctor's Signature: Date:_______ Year______ Month_____ Day

Research Physician Contact Details:

(This page constitutes an essential component of the Study Participant Informed Consent Form. Each "Study Participant Informed Consent Form" must bear the signatures and dates of both the study participant or their legal representative and the study physician to be valid.)

DeepL
Subscribe to DeepL Pro to edit this document.
Visit www.DeepL.com/pro for more information.

Informed Consent Form

Dear Sir/Madam,

We wish to invite you to participate in a clinical study on brain health promotion for the elderly, conducted in collaboration between our hospital and Peking University Health Science Centre. Before deciding whether to join this study, please read the following information as carefully as possible. It will help you understand the nature of the research, its purpose, and the potential benefits, risks, and discomforts it may entail for you.This study has been reviewed and approved by the Ethics Review Committee of Huzhou Third People's Hospital and the Biomedical Ethics Committee of Peking University. It complies with relevant Chinese regulations and ethical principles protecting research participants' rights, including the Declaration of Helsinki.

1. Research Background and Objectives

Mild cognitive impairment (MCI) represents a transitional stage between normal cognitive ageing and dementia, posing a significant threat to global public health.Annually, 5 – 20% of MCI patients progress to dementia, with nearly two-thirds of dementia cases presenting as MCI. In 2018, approximately 50 million older adults worldwide experienced varying degrees of cognitive impairment, projected to increase to 82 million by 2030.In China, over 20% of older adults exhibit MCI, with approximately 6% of these individuals progressing to dementia annually. Non-pharmacological interventions constitute the primary treatment approach for MCI. Research indicates that exercise programmes can reduce the risk of adverse health outcomes in MCI or dementia patients while positively influencing their functional abilities.Research indicates that multicomponent exercise interventions yield superior cognitive outcomes for elderly individuals with MCI, representing a promising non-pharmacological treatment approach characterised by low risk-cost ratios, flexible intervention schedules, and minimal spatial requirements. This facilitates access to treatment for populations in medically underserved regions. Our study aims to develop a brain health intervention model for elderly individuals with mild cognitive impairment, with the objective of enhancing cognitive function.

2. Who will be invited to participate in this study?

Eligible participants

① Individuals aged 60 years or older;

② Screened as MCI patients using the MoCA scale;

③ Capable of walking independently for over five minutes;

④ Leading a predominantly sedentary lifestyle in daily activities (excluding those with regular exercise habits defined as planned, systematic physical activity of moderate intensity for at least 30 minutes per day, 3 days per week, sustained for a minimum of 3 months)

⑤ Self-reported adequate hearing and visual function;

⑥ Obtain informed consent from both the patient and their family.

Exclusion criteria

① Individuals with comorbid mental disorders (including major depressive disorder, intellectual disability, or bipolar disorder);

② Individuals with other neurological impairments;

③ Individuals with neurological or orthopaedic conditions rendering resistance training unsafe (e.g., significant joint pain, osteoporosis);

④ Contraindications for aerobic exercise

⑤ Use of beta-blockers and/or antipsychotic medications;

⑥ Current participation in another intervention programme.

3. How was the study conducted?

All intervention training sessions occur twice weekly for a total of 24 sessions, with the intervention lasting three months. The intervention is delivered in groups of approximately ten participants, with the control group utilising a placebo control approach. Three complimentary health assessments will be conducted at the start, midway, and completion of the intervention, followed by a follow-up assessment three months post-intervention completion.Should you agree to participate, you will be randomly assigned to either the exercise intervention group or the control group. You will not be able to predict or choose which group you are assigned to.

If you meet the inclusion criteria and consent to participate, you will assist the doctor/nurse with the following procedures: Upon enrolment, you will complete questionnaire surveys assessing physical function, quality of life, fatigue, intrinsic capacity (cognitive function, mobility, vitality, sensory function [vision and hearing], depressive symptoms),frailty, and sleep patterns;Assessments will be conducted at the mid-intervention stage (weeks 6-7), post-intervention (weeks 12-13), and 24-25 weeks post-intervention. You will again complete questionnaire surveys with the doctor regarding physical function, quality of life, fatigue, intrinsic capacity (cognitive function, mobility, vitality,vision and hearing sensation, depressive symptoms), frailty, and sleep status.

4. How long will this study last?

This study will conduct a three-month intervention training programme, with assessments scheduled at weeks 6–7 during the intervention period, weeks 12–13 following intervention completion, and weeks 24–25 post-intervention.

5. What are the risks of participating in this study?

Certain questions during the survey may cause you discomfort; you may decline to answer them.Exercise safety concerns may arise during the intervention. Should you experience discomfort or any of the following conditions rendering continued participation inadvisable: (1) exacerbation of existing medical conditions; (2) occurrence of serious adverse events(e.g., falls, fractures, muscle strains, cardiovascular triggers) during the exercise intervention protocol, you may immediately withdraw from the study. The following risk mitigation measures will be implemented:(1) From a safety perspective, enhance participant selection criteria by implementing exercise assessments and screening to exclude individuals unable to walk independently or with contraindications to exercise. Conduct small-scale safety trials prior to large-scale studies to further validate the feasibility of this exercise intervention;(2) Provide relevant exercise safety education to elderly participants in the exercise intervention (including pre-exercise safety reminders such as appropriate clothing and footwear selection, signs requiring immediate cessation of exercise, safety precautions,and situations unsuitable for exercise);(3) Exercise should comprise three phases: preparation,main activity, and cool-down. Incorporating flexibility exercises at both the start and end reduces risks of cardiovascular incidents and accidental injury; (4) Exercise venues should ideally be quiet, with dry, level surfaces and suitable ambient temperatures;(5) It is recommended to provide tables and chairs at the exercise intervention site for participants who feel fatigued or physically overwhelmed to rest; (6) It is recommended that family carers or relatives accompany participants throughout the exercise intervention to enable prompt intervention in case of accidents (despite the recommended movements having a high safety factor and participants being rigorously selected). Any related costs incurred will be borne by the project team; participants will not be required to cover any expenses.

6. What are the benefits of participating in this study?

This brain health promotion programme aims to enhance cognitive function, sleep quality, physical condition, and quality of life among older adults, thereby preventing the progression of dementia. While we cannot guarantee individual benefits from the programme, participation offers opportunities to establish long-term trust with healthcare professionals and cultivate healthy behavioural habits.

7. Is participation in and completion of this study mandatory?

Participation in this study is entirely voluntary. Should you decline, this will have no adverse impact on your current or future medical care. Even after agreeing to participate, you may withdraw at any time by informing the researchers. Your withdrawal will not affect your access to standard medical services.Upon your withdrawal, the researchers will proactively delete any personal information collected. Your relevant data will be securely stored until final destruction, during which time it will not be further used or disclosed.

8. Regarding Study Costs and Compensation

The primary costs associated with this study include equipment fees, material costs, and travel expenses, all of which are covered by the project team's funding. You will not be charged any fees for participating in this research study at .

9. Handling of Research-Related Injuries

Should any accidental injury occur as a result of implementing the research protocol to achieve the study objectives, we shall provide necessary medical treatment and offer compensation or indemnification in accordance with the law.

VIII. Will participating in this study increase my medical expenses?

You will not be charged any fees for participating in this research study. Therefore, this study will not impose any additional financial burden upon you.

IX. Are there alternative treatment options currently available?

Should you choose not to participate in this study, alternative treatment options, such as medication, remain available. Please consult the investigator or your doctor regarding specific treatment plans.

10. Will my information be kept confidential?

Your research data and personal details are strictly confidential. All electronic records will be identified by a research code rather than your name. No personally identifiable information will be disclosed to individuals outside the research team without your explicit consent.

11. Who should I contact if I have questions or difficulties?

For any questions regarding this study, please contact: Hong Chenlu (13141224025). For matters concerning your rights as a participant, you may contact the Ethics Review Committee of Huzhou Third People's Hospital at 0572-2132467. Contact persons: Wen Qiqing, Wang Jun.

Informed Consent Form • Consent Signature Page

Declaration of Consent

1. I have read this informed consent form, and the responsible personnel involved in the project have provided me with a detailed explanation of the trial's purpose, content, risks, and potential benefits.
2. I have discussed and inquired about matters pertaining to this study, and the answers provided have satisfied me.
3. I have had sufficient time to make my decision.
4. I voluntarily consent to participate in the clinical research described herein.
5. Should I withdraw from the trial for personal reasons, I will promptly inform the doctor of any changes in my medical condition.
6. Should any additional treatment become necessary due to changes in my condition, I shall either consult the doctor beforehand or truthfully inform the doctor afterwards.
7. I consent to representatives from the Medicines Regulatory Authority, Ethics Committee, or funding body accessing my research records.
8. I shall receive a signed and dated copy of this informed consent form.
9. I have truthfully disclosed my medical history. Should any information be withheld, I accept full responsibility for the consequences.

Finally, I consent to participate in this clinical study and undertake to comply with all medical instructions.

Signature of Study Participant: Date:_______ _____ _____

Research Participant Contact Number:

Signature of Legal Guardian: Date:_______ Year_____ Month_____ Day

Relationship to Research Participant: Legal Guardian's Telephone:

I confirm that I have explained the details of this study to the research participant, including their rights and the potential benefits and risks, and have provided them with a signed informed consent form.

Doctor's Signature: Date:_______ Year______ Month_____ Day

Research Physician Contact Details:

(This page constitutes an essential component of the Study Participant Informed Consent Form. Each "Study Participant Informed Consent Form" must bear the signatures and dates of both the study participant or their legal representative and the study physician to be valid.)

知情同意书

尊敬的先生/女士：

我们将邀请您参加我院与北京大学医学部合作进行的的一项老年人脑健康促进计划的临床研究。在您决定是否参加这项研究之前，请尽可能仔细阅读以下内容，它可以帮助您了解该项研究内容、为何要进行这项研究以及本研究可能给您带来的益处、风险和不适等。本研究已通过湖州市第三人民医院伦理审查委员会、北京大学生物医学伦理委员会审查，遵从了中国相关法规和赫尔辛基宣言等保护研究参与者权益的伦理原则。

1. 研究背景及研究目的

轻度认知障碍（Mild cognitive impairment，MCI）是介于认知功能正常衰老与痴呆症之间的过渡阶段，严重威胁了全球公众健康。每年有 5%-20%的轻度认知障碍患者发展成为痴呆症，近三分之二的痴呆患者表现为 MCI。2018 年，全球约 5000 万老年人遭受不同程度的认知障碍，据预测，2030 年规模将增加到 8200 万。在中国，有超过 20%的老年人患有 MCI，每年约有 6%的 MCI 老年患者发展为痴呆。非药物干预是 MCI 的主要治疗方法，研究表明运动干预可降低 MCI 或痴呆患者负面健康结局风险并对其功能产生积极影响。研究表明多组分运动干预对 MCI 老年人认知功能效果更佳，是一种有前景的轻度认知障碍非药物治疗方法，其风险成本低、干预时间灵活且空间需求较小，可以帮助医疗资源匮乏地区人群获得治疗机会。我们的研究旨在为轻度认知障碍老年人设计一项脑健康干预模式，以期改善老年人的脑功能。

2. 哪些人将被邀请参加这项研究？

适合人群

① 年龄在 60 岁以上；

② 根据 MoCA 量表筛查为 MCI 患者；

③ 具备独立行走 5 分钟以上的运动能力；

④ 日常生活中为久坐的生活方式（排除有经常性运动习惯的人，经常性运动习惯即至少 3 天/周、30 分钟/天、中等强度的有计划、系统性的身体活动，持续至少 3 个月）

⑤ 自我报告具备一定的听力能力和视觉能力；

⑥ 取得患者及家属知情同意。

不适合人群

① 合并其他精神障碍（包括重性抑郁症、精神发育迟滞、双相情感障碍）；

② 合并其他神经功能障碍；

③ 有神经或骨科合并症导致无法（安全地）进行抗阻训练（如较为严重的关节疼痛、骨质酥松）；

④ 有氧运动禁忌症

⑤ 使用 β-受体阻滞剂和/或抗精神病药物用药；

⑥ 当前正参与另一项干预治疗。

3. 该研究是怎样进行的？

所有的干预训练每周 2 次，共 24 次，干预将持续 3 个月。干预以小组为单位进行干预，每组约 10 人，对照组采用空白对照组的方式。在干预开始时、中期、完成后对您的身体健康进行 3 次免费评估，在干预完成后的 3 个月进行 1 次随访评估。如果您同意参与该项目，您将被随机分配到运动干预组或对照组。您将无法预测或者选择您会被随机分到哪一组。

如果您符合纳排标准并同意参加，您将配合医生/护士完成如下操作： 在入组后，您将配合我们完成身体功能、生活质量、疲劳、内在能力 （认知功能、运动能力、活力、视力和听力感觉、抑郁症状）、虚弱、睡眠状况的量表调查；在干预中期第 6-7 周、干预完成后第 12-13 周和干预完成后的第 24-25 周进行评估；您将再次配合医生完成身体功能、生活质量、疲劳、内在能力 （认知功能、运动能力、活力、视力和听力感觉、抑郁症状）、虚弱、睡眠状况的量表调查。

4. 这项研究会持续多久？

本次研究开展为期 3 个月的干预训练，在干预中期第 6-7 周、干预完成后第 12-13 周和干预完成后的第 24-25 周进行评估。

5. 参加本项研究的风险是什么？

调查过程中的某些问题可能会让您感到不舒服，您可以拒绝回答。干预过程中可能存在运动安全问题，如果您感到不舒服，出现了下列不宜继续进行研究的情况：（1）现患病情加重；（2）严重不良事件的发生，如在完成本研究运动干预方案的过程中发生的不良事件（如跌倒、骨折、肌肉拉伤、心血管疾病诱发等），您可以立即终止研究，并拟采取以下措施控制风险：（1）从安全性角度考虑，提高研究对象的遴选标准，通过运动评定与筛查以排除不能独立行走和存在运动禁忌症者，并在开展大样本试验研究前进行小样本安全性试验，以进一步明确该运动干预的可行性；（2）对参与运动干预的老年人进行相关运动安全教育（包括合适服装和运动鞋的选择等运动前的安全提示、需立即停止运动征兆和安全须知，不适宜进行运动的情况等安全知识）；（3）运动分为准备、运动和整理三个阶段，开始和结束时都要做柔韧性运动可减少心血管意外和意外伤害的风险；（4）尽量选择安静、地面干燥平整、环境温度适宜的场所进行运动；（5）建议在运动干预的现场摆置桌椅，以供自感劳累或者体力不支的老人休息；（6）建议运动干预现场由家庭护理人员或者家人全程陪同，以防发生意外时能够及时处理（尽管推荐的动作安全系数均较高且研究对象是严格筛选的），产生的相关费用由项目组承担，受试者不需要自行承担任何费用。

6. 参加本项研究的获益是什么？

本项脑健康促进计划旨在改善老年人的脑功能、睡眠、身体状况、生活质量等，预防老年痴呆症的发展。我们不能完全保证您能从脑健康促进计划中受益，但您能和医生建立长期信任关系、习得健康行为习惯。

7. 是否一定要参加并完成本项研究？

您是否参加这个研究完全是自愿的。如果您不愿意，可以拒绝参加，这对您目前或未来的医疗不会有任何负面影响。即使您同意参加以后，您也可以在任何时间改变主意，告诉研究者退出研究，您的退出不会影响您获得正常的医疗服务。在您退出之后，研究者将主动删除已经收集的个人信息，研究者将严密保存您的相关信息直至最终销毁，期间不会继续使用或透露这些信息。

8. 关于研究费用和补偿

本研究主要涉及的费用主要包括设备费用、材料费、交通费，各部分费用均来自项目组经费。您参加本

项研究调查不会被收取任何费用。

9. 发生研究相关伤害的处理？

若发生为实现研究目的而执行研究方案造成的意外伤害，我们会提供必要的医疗措施，并依法给予赔偿或者补偿。

八、参加此项研究，是否会增加我的医疗费用？

您参加本项研究调查不会被收取任何费用。因此，本研究不会额外增加您的经济负担。

九、目前是否有其它治疗方法？

若您不参与此研究，您可以选择其他治疗方案，如药物治疗。具体治疗方案请咨询研究者或您的医生。

10. 我的信息会保密吗？

您的研究数据及个人资料的均属保密。您的所有电子材料信息将以研究编码而非您的姓名加以标识。在未获得您的许可之前，任何可识别您身份的信息将不会透露给研究小组以外人员。

11. 如果我有问题或困难，该与谁联系？

如果您有与本研究相关的任何问题，请联系：洪晨璐（13141224025）。如果您有与受试者自身权益相关的问题，可与湖州市第三人民医院伦理审查委员会联系，联系电话：0572-2132467，联系人：温秋卿、王俊。

知情同意书•同意签字页

同意声明

1. 我已经阅读了本知情同意书，项目相关责任人已经将此次试验的目的、内容、风险和受益情况向我作了详细的解释说明。

2. 我已经讨论并询问了有关本研究的相关问题，这些问题的解答令我满意。

3. 我有充足的时间作出决定。

4. 我是自愿同意参加本文所介绍的临床研究。

5. 如果我因个人原因而中途退出，及时将病情变化告诉医生。

6. 如果因病情变化我需要采取任何其他的治疗，我会在事先征求医生的意见，或在事后如实告诉医生。

7. 我同意药品监督管理部门、伦理委员会或课题资助部门代表查阅我的研究资料。

8. 我将获得一份经过签名并注明日期的知情同意书。

9. 我已如实告知病史，如有隐瞒，后果自己承担。

最后，我决定同意参加本项临床研究，并保证遵从医嘱。

研究参与者签名：　　　　　　　　　　日期：_______ 年 _____ 月_____日

研究参与者联系电话：

法定监护人签名：　　　　　　　　　日期：_______ 年 _____ 月_____日

与研究参与者的关系：　　　　　　　　法定监护人电话：

我确认已向研究参与者解释了本研究的详细情况，包括其权利以及可能的受益和风险，并给其一份签署过的知情同意书。

医生签名：　　　　　　　　　　日期：_______ 年______ 月_____日

研究医生联系方式：

（此页为研究参与者知情同意书的必要部分，每一份“研究参与者知情同意书”必须有研究参与者或法定代理人及研究医生的签字和日期，方为有效。）
