# Supplementary material for: Effect of multicomponent exercise intervention on older adults with mild cognitive impairment based on HAPA-TPB theory (MIND-STEP): trial design and baseline data for a randomized controlled trial
Source: Trials. 2026 Mar 23;27:342. doi: 10.1186/s13063-026-09640-4 (PMC13134337; doi:10.1186/s13063-026-09640-4)
Supplement: Supplementary file 2 — Supplementary Material 2: Table: Appendix: All items from the World Health Organisation Trial dataset. [file 13063_2026_9640_MOESM2_ESM.docx]

# Appendix: All items from the World Health Organisation Trial dataset

| **Data category** | **Information** |
| --- | --- |
| **Primary registry and trial identifying number** | ChiCTR2400088301 |
| **Date of registration in primary registry** | 15^th^ August 2024 |
| **Secondary identifying numbers** | n/a |
| **Source of funding** | Medical and Health Science and Technology Program of Zhejiang Province |
| **Primary sponsor** | Medical and Health Science and Technology Program of Zhejiang Province |
| **Secondary sponsor(s)** | n/a |
| **Contact for public queries** | hongchenlu@bjmu.edu.cn |
| **Contact for scientific queries** | hongchenlu@bjmu.edu.cn |
| **Public title** | n/a |
| **Scientific title** | Effect of multicomponent exercise intervention on older adults with mild cognitive impairment based on HAPA-TPB theory: trial design and baseline data for a randomised controlled trial |
| **Countries of recruitment** | China |
| **Health condition(s) or problem(s) studied** | Mild cognitive impairment (MCI) |
| **Intervention(s)** | - Exercise intervention and health education - Health education |
| **Key inclusion and exclusion criteria** | - Inclusion criteria: Participants will be eligible if they meet any of the following conditions: age 60 years or older; screened with MCI using MoCA-B (score range adjusted for education); able to walk independently for at least 5 minutes; sedentary lifestyle (excluding those with a regular exercise routine, which is defined as planned, systematic physical activity of moderate intensity for at least 3 days/week, 30 minutes/day, for at least 3 months); adequate hearing and vision to participate; provided informed consent from the patient and family. - Exclusion criteria: Participants will be excluded if they meet any of the following conditions: diagnosed psychiatric disorders (including major depressive disorder, mental retardation, bipolar disorder); other neurological conditions; neurological or orthopaedic comorbidities that preclude (safe) resistance training (e.g., severe joint pain, advanced osteoporosis); medical conditions contraindicating aerobic exercise; current use of medications that may interfere with beta-blockers and/or antipsychotics; concurrent participation in other structured intervention programs or clinical trials. |
| **Study type** | Randomised controlled trial |
| **Date of first enrolment** | October, 2024 |
| **Target sample size** | 156 participants |
| **Recruitment status** | Ongoing recruitment |
| **Primary outcome(s)** | Cognitive function assessed by the Montreal Cognitive Assessment–Basic (MoCA-B) |
| **Key secondary outcomes** | Quality of life: EuroQol-5D-5L (EQ-5D-5L), Activity Daily of Life (ADL), Pittsburgh Sleep Quality Index (PSQI), intrinsic capacity, frailty, social support and intervention cost and benefit, |
